# Chemical Characterization of *Opuntia ficus-indica* (L.) Mill. Hydroalcoholic Extract and Its Efficiency against Gastrointestinal Nematodes of Sheep

**DOI:** 10.3390/vetsci5030080

**Published:** 2018-09-12

**Authors:** Carolina Santos, Luciano Henrique Campestrini, Douglas Luis Vieira, Izanara Pritsch, Fábio Tomio Yamassaki, Selma Faria Zawadzki-Baggio, Juliana Bello Baron Maurer, Marcelo Beltrão Molento

**Affiliations:** 1 Department of Veterinary Medicine, Federal University of Paraná, Curitiba, PR 80035-050, Brazil; carolinasantos.vet@gmail.com (C.S.); douglasluisvieira@gmail.com (D.L.V.); izanarap@gmail.com (I.P.); 2 Department of Biochemistry and Molecular Biology, Federal University of Paraná, Curitiba, PR 81531-980, Brazil; lcampestrini@gmail.com (L.H.C.); japa_vascaino@yahoo.com.br (F.T.Y.); sfzb@ufpr.br (S.F.Z.-B.); jumaurer@ufpr.br (J.B.B.M.)

**Keywords:** cactus pear, phenolic compounds, hatchability, antiparasitic

## Abstract

*Opuntia ficus-indica* (L.) Mill. is a xerophylous plant that originated in tropical and subtropical America. This plant is popularly known in Brazil as “palma forrageira” (cactus pear) and plays a fundamental role in animal nutrition, mainly in the Northeastern semi-arid region of the country. The plant has several uses since it presents bioactive compounds that confer biological and pharmacological properties. In this context, the cactus pear can also be considered a potential product to combat parasite infections. The objective of this study was to chemically characterize the *O. ficus-indica* hydroalcoholic extract (OFIEOH) and to determine its efficacy against gastrointestinal parasites using in vitro tests. Initially, the hydroalcoholic extract from cladode peels of *O. ficus-indica* was produced by maceration for 21 days. For the chemical characterization, colorimetric dosages were performed for carbohydrates, proteins, phenols and condensed tannins. Liquid chromatography coupled to mass spectrometry/electron spray ionization (LC-MS/ESI) was used to characterize the polyphenolic profile of the OFIEOH extract. Fifteen compounds were identified in the OFIEOH extract, such as methyl, glycosylated and aglycone quercetin derivatives and aglycone and glycosylated kaempferol derivatives. Tri-glycosylated methyl quercetin derivatives were the main compounds identified. In vitro egg hatch (EHT) and larval migration tests (LMT) were used in a range of concentrations of OFIEOH from 12.5 to 100 mg/mL for EHT and 12.5 to 200 mg/mL for LMT. In addition, the LMT was used to test ivermectin (IVM) (from 11.4 to 57.1 µM), associated with the inhibitory concentration of 50% (IC_50_) for OFIEOH. The combination of OFIEOH (12.5 to 200 mg/mL) plus the IC_50_ of IVM was also tested. The efficacy of OFIEOH alone varied from 19.33 to 90.0% using the EHT. The LMT revealed an efficacy of 5.78 to 77.26% for the extract. Both tests showed a concentration-dependence inhibitory effect. We found a drug-extract antagonistic neutralizing effect when doses of IVM were added to OFIEOH (maximum efficacy of 73.78%), while a positive additive effect was observed when OFIEOH was added to the IC_50_ of IVM (IC_50_ of 82.79 for OFIEOH alone against an IC_50_ of 55.08 of OFIEOH + IVM). The data from this work indicate that OFIEOH alone may be considered as a suitable ecofriendly product to control gastrointestinal parasites of sheep, offering a more holistic approach to improve animal farming and welfare. The drug-extract interaction is also a promising therapeutic alternative, reducing the final dose to the host, with an optimum combination effect.

## 1. Introduction

In recent decades, the use of medicinal plants in human and veterinary medicine has been expanding as an aid to the prevention and treatment of diseases, caused by bacteria or parasites [1,2]. In Brazil, the use of medicinal plants for such purposes presents strong influence from the Native indigenous people, African and European culture [3,4]. Among the various plants studied for pharmacological purposes, *Opuntia ficus-indica* (L.) Mill. (Cactaceae) is a plant popularly known in Brazil as “palma forrageira” (cactus pear). It is widely known because of its nutritional and medicinal usage [5,6]. This cactus was introduced to Brazil during the end of the 19th century with the objective to produce carmine dye [7]. It was after a severe drought in 1932 that this plant was discovered as an excellent forage alternative for animals [8]. The vegetative parts, called cladodes, are adaptations of the stems that replace the leaves in their photosynthetic function and can store large amounts of water that is used by animals [6]. This high-water content property is considered an important quality to semi-arid regions of Brazil, as it may be the only alternative water supply for animals during long drought periods [9]. The cactus can also be considered a potential product for veterinary purposes, being a source of a new anti-parasitic compound [10,11].

Distinct biological activities of the cactus pear including hypoglycaemic, antioxidant, antimicrobial, antiulcer and anti-inflammatory have been described [5,6,12]. These biological properties usually have been related to the phenolic content profile present in the plant [5,13,14]. Despite the knowledge of the various properties of the constituents of the cactus pear, few studies have been carried out to determine its anthelminthic activity and the compounds that may be involved with these effects [11]. According to reference [11], the anthelminthic activity expressed by this plant is attributed to the sum of the effects of phytochemicals such as saponins flavonoids, plant pigments, and tannins. However, as mentioned above, there is a need to further investigate some of these gaps to enable the use of *O. ficus-indica* beyond its nutritional potential, making it an alternative resource to the control of parasite infections.

Due to the importance of *O. ficus-indica* to animals in Brazil and other regions [6,8], the aim of this work was to perform a phytochemical characterization of the hydroalcoholic extract of *O. ficus-indica* cladode peels (OFIEOH), as well as to perform in vitro egg hatch and larval migration tests to determine its efficacy against gastrointestinal nematodes of sheep. The OFIEOH was also used in combination with ivermectin (IVM), a product from the macrocyclic lactone family, to determine potential drug-extract interaction.

## 2. Materials and Methods

### 2.1. Plant Material and Preparation of the Hydroalcoholic Extract of the Cladode Peels of O. ficus-indica

The cactus pear cladodes were collected in September 2015 at Rancho Colorado Farm, in the city of Macaíba, state of Rio Grande do Norte, Northeast region of Brazil. The plant was identified at the herbarium of the Federal University of Paraná (voucher number: 85365).

Initially, the cladodes of the plants were separated into distinct parts: peel and pulp. The peels (2.1 kg) were cut into different sizes and subsequently dried in an oven at 40 °C for 72 h. To prepare the hydroalcoholic extract, 191.5 g of peels, corresponding to the entire dried material and 1 L of 80% ethanol were conditioned in a glass flask protected from light. The mixture was kept under maceration at 4 °C for 21 days. During this period, extractive solvent exchanges were performed on days 7 and 14. On day 21, the extract was pooled, filtered and evaporated on a rotary evaporator at 40 °C. After that, the extract was freeze-dried, corresponding to the OFIEOH. OFIEOH presented a yield of 9% in relation to the dry plant material (dry cladode peels). OFIEOH was resuspended in water for the in vitro tests.

### 2.2. Total Carbohydrate Content

The total carbohydrate content was determined by a previously described method [15]. d-Glucose (Sigma-Aldrich, St. Louis, MO, USA) was used to prepare a standard curve with concentrations from 4 to 40 µg/mL (R^2^ = 0.9958). OFIEOH was solubilized in H_2_O at 1 mg/mL and 40 µL of this solution was transferred to 2 mL microtubes in triplicate. After that, 40 μL of 5% phenol (*m*/*v*) was mixed with the solutions. Subsequently, 200 μL of concentrated sulfuric acid (H_2_SO_4_) was added. The solution was heated at 100 °C for 10 min and then cooled in ice-bath. After that, 200 μL of the solution was transferred to a 96-well reading plate and the absorbance was measured, at 490 nm, on a microplate reader spectrophotometer EPOCH-ELX800 (BioTek Instruments, Winooski, VT, USA). In this assay, carbohydrates react with sulfuric acid undergoing dehydration and complexation with phenol, generating a yellow-orange coloration. The assay was performed in triplicates.

### 2.3. Total Protein Content

The protein content was determined using the Coomassie Brilliant Blue G-250 dye (Sigma-Aldrich, St. Louis, MO, USA), as previously described [16]. First, the standard dose curve was set with bovine serum albumin (Sigma-Aldrich, St. Louis, MO, USA) with a sensitivity limit from 4 to 40 µg/mL (R^2^ = 0.9952). Subsequently, OFIEOH was solubilized in distilled H_2_O at 1 mg/mL concentration. Aliquots of 40 μL of OFIEOH were transferred to microtubes, then 400 μL of the Bradford reagent was added. After shaking, 200 μL was transferred to a 96-well microplate and the absorbance was measured at 595 nm. All samples were analyzed in triplicate.

### 2.4. Total Phenolic Content

The total phenolic content was determined by the Folin–Ciocalteu method [17]. The standard curve was prepared using solutions of gallic acid at concentrations of 1, 5, 10, 20, 30, 40 and 50 µg/mL (R^2^ = 0.9516). Aliquots (250 µL) of OFIEOH at 1 mg/mL concentration in H_2_O were added to test tubes. Next, 1.5 mL of the Folin-Ciocalteu reagent [10% (*v*/*v*) in distilled H_2_O] obtained from Chromato (São Paulo, Brazil) was added. The tubes were shaken, and then 1 mL of 7.5% (*v*/*v*) sodium carbonate (Na_2_CO_3_) was added to the solutions. After heating in a water bath at 50 °C for 5 min, aliquots of 200 μL were transferred to a 96-well microplate. The absorbance was measured at 760 nm. The principle of the method is that the reaction between the Folin-Ciocalteu reagent and phenolic compounds results in the formation of a blue complex. This reaction was favored by an alkaline pH medium, obtained by the addition of carbonate. All samples were analyzed in triplicate.

### 2.5. Condensed Ttannins Content

The dosage of condensed tannins was performed using sulfuric vanillin as a reagent, by a method described elsewhere [18]. Aliquots of 1 mL of OFIEOH at 5 mg/mL concentration in distilled water were mixed with 2 mL of 2% (*v*/*v*) sulfuric vanillin solution. The mixture was kept in a water bath at 20 °C for 5 min. Aliquots of 200 μL were transferred to a 96-well microplate and the absorbance was measured at 500 nm. Commercial epicatechin (Sigma-Aldrich, St. Louis, MO, USA) was used for the standard curve, and the concentrations ranged from 1.25 to 30 μg/mL (R^2^ = 0.9996). All samples were tested in triplicate.

### 2.6. Liquid Chromatography Coupled to Mass Spectrometry/Electron Spray Ionization (LC-MS/ESI)

LC-MS/ESI was used to characterize the polyphenolic profile of the OFIEOH extract. The analysis was conducted using an Agilent 1200 series coupled with a triple quadrupole MS series 6400 (Santa Clara, CA, USA). Separation was performed using a C18 reverse-phase column (150 × 2.1 mm, 5 μm) Shim-Pack CLC-ODS (Shimadzu, Kyoto, Japan) at 25 °C. The mobile phase for the HPLC was prepared with acetonitrile (99.98% purity) from J.T. Baker (Xalostoc, Mexico), and Merck’s formic acid (≥99%). The mobile phase consisted of 0.5% formic acid in water (solvent A) and 0.5% formic acid in acetonitrile (solvent B) using the following gradients: 0–10 min: 10% B; 10–20 min: 10–30% B; 20–30 min: 30–50% B; 30–32 min: 50% B; 32–38 min: 50–10% B; 38–48 min: 10% B [19]. The flow rate was 1.0 mL/min and the injection volume was 5 μL. Before the analysis with LC-MS/ESI, 1 mg/mL of OFIEOH extract was eluted through the C18 cartridges (Varian, Palo Alto, CA, USA) to remove low-molecular-weight compounds. The parameters for ESI were as follows: nebulizer at 50 psi, 4500 V, arrest gas (N2) flow at 11 L/min, and an ionization temperature of 350 °C. The ion trap mass spectrometer was operated in a negative ion mode with a full scanning range from *m*/*z* 150 to *m*/*z* 1000.

### 2.7. Fecal Sample Collection and Eggs Retrieval

Prior authorization for the use of animals was obtained from the Ethical Committee for the Use of Animals of the Federal University of Parana (UFPR), under the number 008/2016. Fecal samples were obtained from 10 naturally infected sheep of the Sheep and Goats Research Unit, located at the Canguiri Farm of UFPR. The feces were collected directly from the rectum of previously selected sheep, with fecal egg counts higher than 1600 eggs. The eggs were cleaned from the fecal material according to an adaptation of a method proposed by reference [20], where 20 g of feces were initially weighed, macerated and homogenized in warm water at 28 °C. Then, the samples were washed and filtered through 400, 250, 150, 75 and 25 μm aperture sieves in order to recover the eggs. In the last sieve, the eggs were washed with distilled water and centrifuged in 50 mL Falcon tubes at 140× *g* for 5 min. The eggs were recovered and counted under an optical microscope SM-LUX (Ernst Leitz GmbH, Wetzlar, Germany). We performed a culture from a pool of feces and identified 88% of *Haemonchus contortus*, followed by 12% of *Trichostrongylus* spp.

### 2.8. Egg Hatch Test (EHT)

The test was performed according to the methodology described by reference [20], with modifications. After washing, recovery, and quantification of the eggs, approximately 100 eggs in suspension were added per well into a 24-well plate and mixed with the OFIEOH prepared in distilled water at final concentrations of 3.125, 6.25, 12.5, 25, 50, and 100 mg/mL, in a total volume of 1 mL. We performed the experiment using triplicate samples for each concentration. The plates were incubated for 24 h at 27 °C and egg counts were used to calculate the egg hatch percentage using the following formula: Egg hatchability (%) = L1/(eggs + L1) × 100, where L1 corresponds to first stage larvae. The larval count was done on an inverted microscope. The average number of hatched eggs at each concentration was transformed into a percentage by the GraphPad Prism 7 program. The positive control was made using IVM (114.3 µM), solubilized in hot water. The negative control was prepared with distilled water.

### 2.9. Larval Migration Test (LMT) and Extract-Drug Interaction

The LMT was performed to according the methodology described by reference [21] and modified by reference [22], with adaptations. Initially, the sheath was removed from the infective third-stage larvae (L3) using 0.3% (*v*/*v*) sodium hypochlorite for 1 h. Subsequently, L3 were washed three times by centrifugation (at 140× *g* for 5 min) with distilled water and then quantified to add in 24-well plates (approximately 200 larvae/well). The concentrations of OFIEOH were 12.5, 25, 50, 100, 150, and 200 mg/mL. Each concentration was tested in triplicate. The first incubation was for 6 h at 27 °C. After that, all solutions containing the larvae, extract, positive, and negative control were transferred to previously prepared apparatus. These apparatuses were incubated for the second time, at 27 °C, under a 150 MHz light source to stimulate larval motility. After 18 h, the solution was transferred to 50 mL tubes and centrifuged at 140× *g* for 2 min. A 2 mL aliquot was transferred from the decantation of each well into a 24-well plate. Larval counts were performed under optical microscopy (10×). The average number of migrating L3 at each concentration was transformed into a percentage by the GraphPad Prism 7 program. We used IVM (114.3 µM) as a positive control and the negative control was prepared with distilled water.

The LMT was also used to evaluate the efficacy of the extract in combination with IVM. For both, the inhibitory concentration of 50% (IC_50_) was used. Initially, concentrations from 12.5 to 200 mg/mL of OFIEOH were tested with the addition of 25.7 µM of IVM. In a parallel test, concentrations from 11.4 to 57.1 µM of IVM were added to 82.80 mg/mL of OFIEOH.

### 2.10. Statistical Analysis

The results of the in vitro tests (antiparasitic efficacy) were evaluated through analysis of variance (ANOVA) and were considered statistically significantly different when presented with a probability of occurrence of the null hypothesis of less than 5% (*p* < 0.05). The calculation of the IC_50_ was performed by adjusting the non-linear regression data using normal and logistic distributions for the extracts and IVM. All analyses were performed using the GraphPad Prism 7 program.

## 3. Results and Discussion

### 3.1. Colorimetric Dosages of the OFIEOH of Cladode Peels from O. ficus-indica

Regarding the colorimetric dosages of total carbohydrate and protein, the OFIEOH presented 250 mg of d-glucose equivalent/g dry extract (25%) and 2 mg of bovine albumin equivalent/g dry extract (0.2%), respectively. According to reference [5], the carbohydrate and protein contents found in cladodes in natura were from 3 to 7% and from 0.5 to 1%, respectively. As expected, the composition of the plant material may vary according to the edaphoclimatic conditions, cultivation method, the season of the year, the origin of collection, and age of the plant [23].

The total phenol content was 520 mg of gallic acid equivalent/g of dry extract. These values were higher than those found by reference [24], which verified concentrations of 180.3 mg/g from the ethanolic extract of *O. ficus-indica* var. *saboten*. The differences between the observed concentrations may be due to the extraction methodology, since factors such as the temperature may positively influence the extraction process [25].

Regarding the content of condensed tannins from the OFIEOH, it corresponded to 1.20 mg of epicatechin equivalent/g of dry extract concentration. Of note, it is important to mention that despite the significant phenolic content, other components that were not tannins were predominantly present in OFIEOH. Other studies with *O. ficus-indica* extracts described variations of tannin contents from 1.2 to 3.2 mg of proanthocyanidins/g dry extract [23]. According to reference [14], using extracts from fruit skin and seeds of *Opuntia* spp., the authors found that tannins were the main phenolic compounds, representing almost 50% of the total contents. Similarly, according to reference [11], it was verified that large amounts of tannins, as well as alkaloids and flavonoids were present in extracts from crude cladodes and ethanolic extracts of fruits of *O. ficus-indica*. This variation demonstrated that different portions of the plant could also affect the phytochemical composition. As mentioned before, different cultivation and climatic conditions can influence the composition of plant constituents [23]. This information also underscores the importance of studies using different fractions of plants, with the objectives to improve their activity and to identify their major components.

### 3.2. Characterization of Phenolic Profile of OFIEOH Extracted by LC-MS/ESI

**Figure 1:**
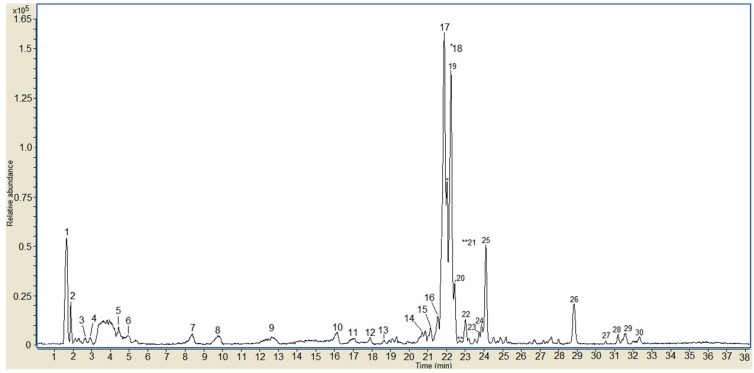
LC-MS/ESI chromatogram of OFIEOH from *Opuntia ficus-indica*. Numbers in the chromatogram refer to the compounds described in Table 1.

To characterize the contents of the phenolic compounds of OFIEOH extract, the sample was analyzed by LC-MS-ESI. Polyphenolic compounds were identified by comparing the resultant data with the available data [26,27,28,29,30]. The LC-MS-ESI profile of the OFIEOH extract (Figure 1) presented 30 peaks. From these, 15 peaks were identified as indicated in Table 1. The full mass spectra of the peaks showed deprotonated molecules [M − H]^−^ at *m*/*z* 447, 300, 739, 769, 755, 609, 593, 623, 315, 299 and 297 (Table 1). The mass spectra are presented in Figure S1. The phenolic compounds identified corresponded to methyl, glycosylated and aglycone quercetin derivatives and aglycone and glycosylated kaempferol derivatives. The main compounds (peaks 17, 18, and 19) (molecular ion at *m*/*z*: 769), present in the OFIEOH, were identified as tri-glycosylated methyl quercetin derivatives, with a sugar portion consisting of two deoxyhexose and one hexose. According to the literature, these derivatives can be isorhamnetin-3-*O*-Rha-7-*O*-rutinoside and/or rhamnetin-3-*O*-Gal-6-*O*-Rha-3-*O*-Rha or other tri-glycosylated methyl quercetins [26,28,30]. Other quercetin derivatives, presenting molecular ions at *m*/*z* 300 (peak 14) and 315 (peak 29), were identified as an aglycone form of quercetin and methyl-quercetin (as narcissin), respectively [30]. Tri-glycosylated quercetin (such as quercetin-3-neohesperidoside-7-rhamnoside) (with two deoxyhexose and one hexose) (peaks 20 and 21, *m*/*z* 755) and di-glycosylated quercetin (such as rutin), with one deoxyhexose and one hexose (peak 22, *m*/*z* 609), were also identified. Peaks 24 and 25, with *m*/*z* 623, were identified as di-glycosylated methyl quercetin, with one deoxyhexose and one hexose as the sugar moieties [27,28]. In relation to kaempferol derivatives, we identified kaempferide (*m*/*z* 299, peak 29) and mono-glycosylated kaempferol with a hexose as the sugar component (*m*/*z* 447, peak 4) [26]. Peak 16 (*m*/*z* 739) suggested the presence of tri-glycosylated kaempferol (as nicotiflorin), with its sugar portion consisting of two deoxyhexose and one hexose. The detected ion at *m*/*z* 593 was identified as kaempferol-3-*O*-rutinoside, where the sugar portion consisted of one hexose and one deoxyhexose [30]. In addition, the presence of a mono-glycosylated acetyl phenyl derivative (peak 29) with *m*/*z* 298, was suggested. Other studies focusing on the characterization of phenolic compounds of *O. ficus-indica* also described the presence of rutin, narcissin and nicotiflorin in cladode extracts [5,13,27,28,29,30].

Phenolic compounds fall into several categories such as simple phenols, phenolic acids (derivatives of benzoic and cinnamic acids), coumarins, flavonoids, stilbenes, condensed, and hydrolysates tannins and lignins [31]. Flavonoids represent one of the major classes of plant phenols and are found as many variations such as flavonols, flavones, flavanones, flavanols or catechins, anthocyanins, isoflavones, chalcones and dihydrochalcones [32]. From the subclass of the flavonols, quercetins (3,3′,4′,5,7-pentahydroxyflavone) can be found in the aglycone forms or, more commonly, in the form of glycosides and/or as methylated or acylated derivatives [32]. Quercetin is known for having a potent antioxidant and anti-inflammatory activity and it has been associated with the prevention and therapy of cardiovascular diseases and cancer [33]. Although polyphenolic compounds, such as tannins, have anthelmintic effects, the antiparasitic capacity of quercetins has been recently studied [34,35]. According to reference [36], the ethanolic extract of *Artemisia campestris*, a plant rich in quercetin derivatives, was evaluated using in vitro tests and presented 100% inhibition of the egg hatch rate and larval motility of *H. contortus* at a dose of 2 mg/mL of the extract. Similarly, reference [37] reported that quercetin and kaempferol present in the extract of *Bridelia ferruginea* bark caused the mortality of *Fasciola gigantica* and *Taenia solium* larvae in less than 62 min., demonstrating its use as an anthelmintic plant.

### 3.3. Egg Hatch Test (EHT) and Larval Migration Test (LMT)

**Figure 2:**
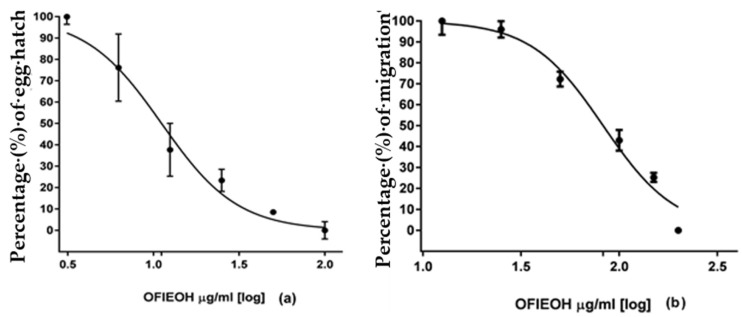
Effect (percentage and standard deviation) on (**a**) egg hatch and (**b**) larval migration of *Opuntia ficus-indica* essential oil (OFIEOH) against gastrointestinal nematodes of sheep.

The results of the EHT and LMT were concentration-dependent (Figure 2a,b). For the EHT, the concentration of 100 mg/mL (Table 2) and for the LMT, the concentrate on of 200 mg/mL (Table 3) presented the highest effect, similar to the positive control (Table 2 and Table 3).

Positive inhibition of the egg hatch rate was also verified by reference [11], evaluating a cladode aqueous extraction of *O. ficus-indica*. In their study, the authors found an efficacy of 93% using 100 mg/mL of the extract. The value was very similar to the present study (90%). In the same study, the authors found the cladode extract to be even more effective using the LMT. We found very different results (IC_50_ of 82.79 mg/mL), in comparison with reference [11] using the larval inhibition assay (IC_50_ of 0.74 mg/mL) but using twice as much of the extract. This may have some therapeutic implications but we want to perform new experiments to improve the present data.

In vitro tests of OFIEOH demonstrated the potential of the plant as a natural antiparasitic, although the tests were performed with the total extract and thus, it would be difficult to attribute any parasite control effect to a class or component of the described phytochemicals. However, analyzing the composition of the extract, we verified that the antiparasitic effects may be due to the large fraction of phenolic compounds and more specifically the quercetin derivative. In contrast, reference [11] used the EHT and LMT in the partitioned extracts from cladodes and found a higher efficacy, probably from the tannin-rich components.

Tannin-rich plants have been used against gastrointestinal parasites and have highlighted the direct effects of tannins by promoting structural alterations in the parasites, reducing the development of the larval stages and by compromising the fertility of females and egg integrity (hatchability) [34,35]. However, the high concentration of this component was not evidenced in our study. Thus, the findings of the present work suggest that other phenolic compounds, such as flavonoids, may also exert antiparasitic activity, alone or in combination. According to reference [32] and reference [36], there is a possibility of synergistic effects among compounds, such as quercetin derivatives, which was verified by the high concentration used in our study. The synergistic effect among plant ingredients was also verified by reference [38] that evaluated the combination effect of different flavonoid compounds through larval tests.

### 3.4. Larval Migration Test (LMT) and Extract-Drug Interaction

The LMT was used to test the extract-drug interaction due to the robustness of the in vitro L3 motility test. The results of the inhibition of L3 migration using the combination of IVM and the IC_50_ of OFIEOH are shown in Table 4. The results of the inhibition of L3 migration using the association of OFIEOH and the IC_50_ of IVM are shown in Table 5.

As shown above, the efficacy of the different compounds has also evidenced a concentration-dependent effect, as the best efficacy values were found with the highest concentrations of both treatments. In this sense, the concentration of 57.1 µM of IVM plus 82.80 mg/mL of OFIEOH, and 200 mg/mL of OFIEOH plus 25.7 µM of IVM, had the highest effect on L3 motility (Table 4 and Table 5).

The interest in using anthelmintic combinations arises from the increasing problem of parasite resistance [39] and, thus, the association of different chemical groups may allow for a more effective control, delaying the development of resistance [40,41]. However, unfavorable interactions between the active compounds are possible and should be considered [42].

**Figure 3:**
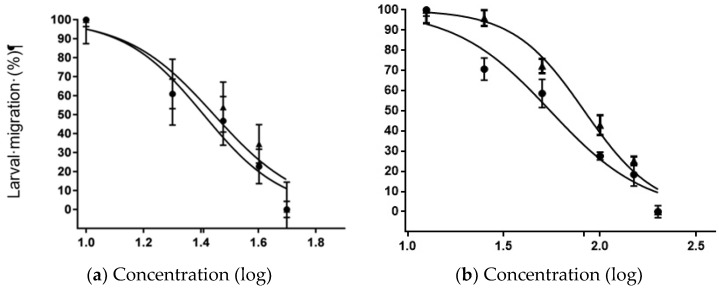
Concentration-dependent ((µg/mL(log)) effect curves for the larval migration test (%) using nematodes of sheep, with the interaction of (**a**) ivermectin alone (triangle) or in combination with the IC_50_ of OFIEOH (circle); and (**b**) OFIEOH alone (triangle) or in combination with the IC_50_ of ivermectin (circle), obtained from the peels of *Opuntia ficus-indica*. IC_50_ = Inhibitory concentration of 50% of larvae.

Analyzing the interaction curves between IVM and OFIEOH, we observed that the inclusion of the IC_50_ of OFIEOH (IC_50_ 25.65 mg/mL) and the different doses of IVM (IC_50_ 27.86 mg/mL) did not contribute to the efficacy of the extract, showing a neutral antagonist effect (Figure 3a). The mechanism of interaction, in this case, was probably by limiting their independent intrinsic activity, with no addition of the individual efficacy (superimposed curves). The addition of the IC_50_ of IVM plus the different doses of OFIEOH (IC_50_ 55.08 mg/mL) presented a statistically significant difference (*p* < 0.05) additive effect, when compared to the effect of the extract alone (IC_50_ 82.79 mg/ml), increasing the efficacy of the extract on the larvae (Figure 3b). Although, in general, experimental data on plant-drug interactions are limited [43], similar to our study, a synergistic effect was verified by reference [44] that evaluated the effect of drug interactions between a natural product (dehydrated garlic—*Allium sativa*) and two commercially available anthelmintics (levamisole and IVM), against sheep nematodes. Our results showed that OFIEOH plus IVM increased L3 inhibition by 1.5 times. A synergistic effect was also observed by reference [22] when using IVM in combination with verapamil, a calcium channel blocker, with LMT and [45] in vivo, against *H. contortus* naturally infected sheep. The effect of the combination of phenolic compounds and/or flavonoids with commercial drugs to control gastrointestinal parasites of livestock still has to be extensively tested to secure maximum animal safety.

## 4. Conclusions

The OFIEOH from cladode peels is composed mostly of phenolic compounds belonging to the flavonoid class, including aglycone, methyl and glycosylated quercetin derivatives, aglycone, and glycosylated kaempferol derivatives. The tri-glycosylated methyl quercetin derivative was the main compound identified in the OFIEOH. The extract had a concentration-dependent effect, in which a clear gradual reduction of the egg hatch rate and larval motility was observed. The interaction effects between OFIEOH alone and when it was used in different concentrations with the addition of LD_50_ of IVM was antagonistic neutral. The results of IVM alone or in association with LD_50_ of OFIEOH showed an additive effect, improving the absolute efficacy by 1.5 times. Although the extract itself demonstrated to have a promising chance as a biofriendly antiparasiticide, new studies should be carried out to maximize its effectiveness, including the positive outcome of drug combinations, with the objective to reduce the amount of product that would be given to the final host.

## Figures and Tables

**Table 1:** Polyphenol compounds of *Opuntia ficus-indica* essential oil (OFIEOH) identified by LC-MS/ESI.

Peak	R_t_ (min)	[M − H]^−^ (*m*/*z*)	Identified and Suggested Compounds
5	4.4	447	mono-glycosylated kaempferol
14	20.1	300	quercetin
16	21.5	739	tri-glycoslylated kaempferol
17	21.7	769	tri-glycosylated methyl-quercetin derivative I
18	21.9	769	tri-glycosylated methyl-quercetin derivative II
19	22.0	769	tri-glycosylated methyl-quercetin derivative III
20	22.2	755	tri-glycosylated quercetin I
21	22.4	755	tri-glycosylated quercetin II
22	23.0	609	di-glycosylated quercetin (Rutin)
23	23.7	593	di-glycosylated kaempferol
24	23.9	623	di-glycosylated methyl-quercetin I
25	24.1	623	di-glycosylated methyl-quercetin II
26	28.8	315	methyl-quercetin
29	31.6	299	kaempferide (Kaempferol 4’-methyl ether)
30	32.3	297	mono-glycosylated acetyl phenyl derivative

Details of the LC analysis conditions see in the material and methods section. R_t_: Retention time. Peaks 1 to 4; 6 to 13, 15; 27 and 28 were not identified.

**Table 2:** Percentage (±standard deviation) of inhibition of egg hatch of gastrointestinal nematodes in the presence of *Opuntia ficus-indica* essential oil (OFIEOH) from cladode peels. The IC_50_ was based on the extract calculations and ivermectin was used as positive control.

OFIEOH (mg/mL)	Inhibition of Egg Hatch (%)
100	90.00 ± 3.04
50	80.67 ± 0.96
25	70.67 ± 3.94
12.5	61.00 ± 9.39
6.25	33.67 ± 11.92
3.125	19.33 ± 2.74
H_2_O	5.33 ± 2.98
ivermectin (114.3 µM)	90.67 ± 3.63
IC_50_	11.15 mg/mL

**Table 3:** Percentage (±standard deviation) of inhibition of larval migration of gastrointestinal nematodes in the larval migration test in the presence of *Opuntia ficus-indica* essential oil (OFIEOH) from cladode peels. The IC_50_ was based on the extract calculations and ivermectin was used as positive control.

OFIEOH (mg/mL)	Inhibition of Larval Migration
200	77.26 ± 0.66
150	59.21 ± 1.77
100	49.57 ± 4.22
50	25.63 ± 2.88
25	8.66 ± 3.11
12.5	5.78 ± 5.33
H_2_O	1.08 ± 4.44
ivermectin (114.3 µM)	87.00 ± 4.66
IC_50_	82.79 mg/mL

**Table 4:** Percentage (±standard deviation) of inhibition of migration of gastrointestinal nematodes using the larval migration test with the interaction of ivermectin and the concentration equivalent to the IC_50_ of *Opuntia ficus-indica* essential oil (OFIEOH).

Concentration of IVM (µM) + OFIEOH (mg/mL)	Inhibition (%)
57.1 + 82.80	73.78 ± 6.11
47.7 + 82.80	53.85 ± 4.11
34.3 + 82.80	42.66 ± 5.53
22.8 + 82.80	38.11 ± 6.68
11.4 + 82.80	16.08 ± 1.33
H_2_O/Control	0.70 ± 2.22
IC_50_ IVM	25.75 µg/mL

**Table 5:** Percentage (±standard deviation) of inhibition of migration of gastrointestinal nematodes using the larval migration test with the interaction of *Opuntia ficus-indica* essential oil (OFIEOH) and the concentration equivalent to IC_50_ of IVM.

Concentration of OFIEOH (mg/mL) + IVM (µM)	Inhibition (%)
200 + 25.7	62.49 ± 2.11
150 + 25.7	45.82 ± 4.22
100 + 25.7	37.48 ± 1.33
50 + 25.7	9.35 ± 5.33
25 + 25.7	3.09 ± 7.33
12.5 + 25.7	1.53 ± 4.11
H_2_O/Control	0.35 ± 3.33
IC_50_ OFIEOH	82.79 mg/mL

## Supplementary Materials

The following are available online at http://www.mdpi.com/2306-7381/5/3/80/s1, Figure S1: MS spectra of polyphenol compounds in *O. ficus-indica* cladodes.
