# Supplementary material for: Chemical Characterization of Opuntia ficus-indica (L.) Mill. Hydroalcoholic Extract and Its Efficiency against Gastrointestinal Nematodes of Sheep
Source: Vet Sci. 2018 Sep 12;5(3):80. doi: 10.3390/vetsci5030080 (PMC6163712; doi:10.3390/vetsci5030080)
Supplement: Supplementary file 1 [file vetsci-05-00080-s001.pdf]

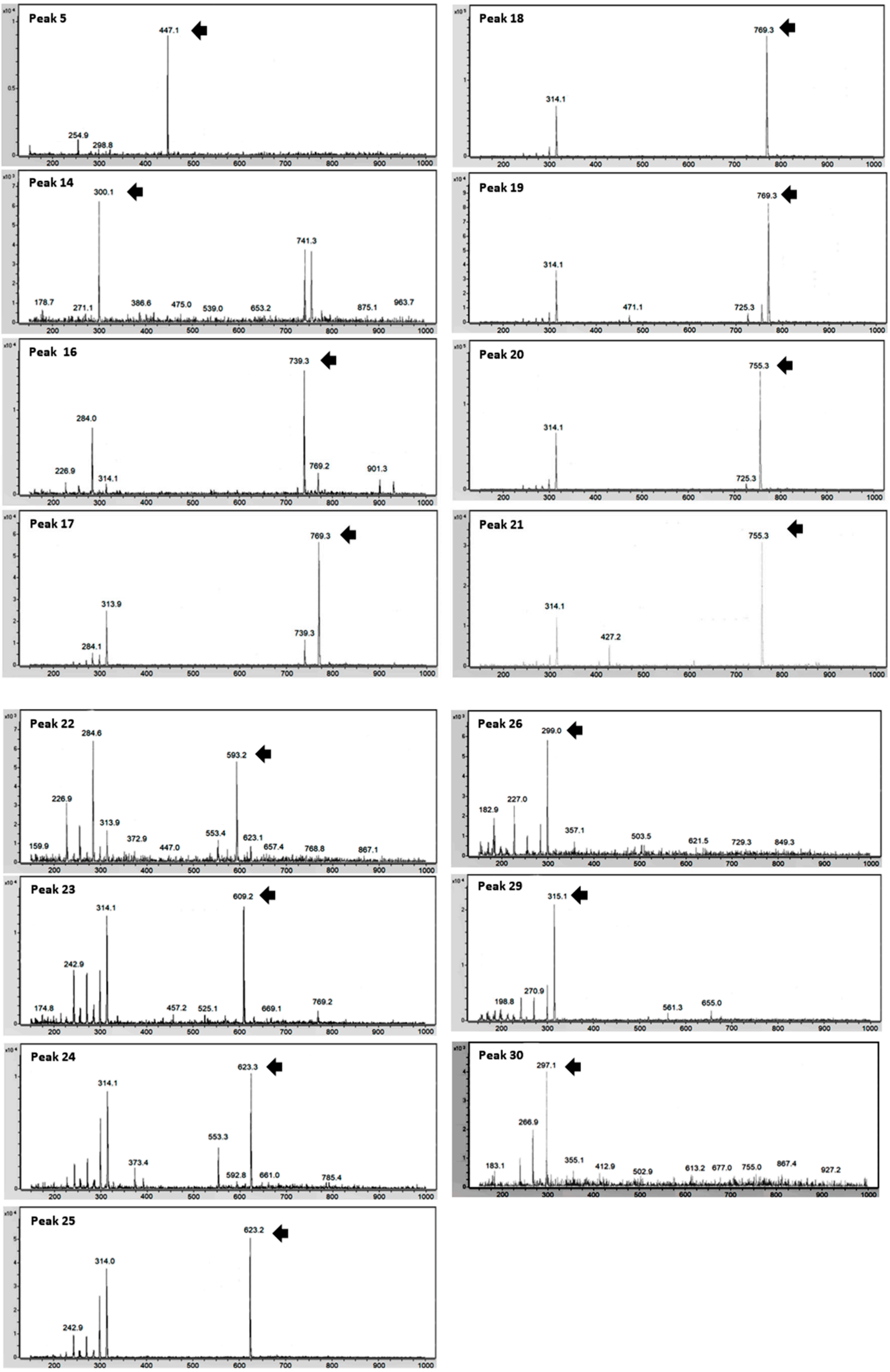

Figure S1. MS spectra of polyphenol compounds in *O. ficus-indica* cladodes. For details on the conditions of the LC-MS/ESI analysis, see the materials and methods section.
